# Molecular Cytogenetic Characterization of the Sicilian Endemic Pond Turtle *Emys trinacris* and the Yellow-Bellied Slider *Trachemys scripta scripta* (Testudines, Emydidae)

**DOI:** 10.3390/genes11060702

**Published:** 2020-06-25

**Authors:** Rita Scardino, Sofia Mazzoleni, Michail Rovatsos, Luca Vecchioni, Francesca Dumas

**Affiliations:** 1 Department of Biological, Chemical and Pharmaceutical Sciences and Technologies (STEBICEF), University of Palermo, 90100 Palermo, Italy; rituccia1989@gmail.com (R.S.); luca.vecchioni@unipa.it (L.V.); 2 Department of Ecology, Faculty of Science, Charles University, 12844 Prague, Czech Republic; sofia.mazzoleni@natur.cuni.cz (S.M.); mirovatsos@gmail.com (M.R.)

**Keywords:** *Emys trinacris*, FISH, karyotype, rDNA, telomeric sequences, *Trachemys scripta scripta*

## Abstract

Turtles, a speciose group consisting of more than 300 species, demonstrate karyotypes with diploid chromosome numbers ranging from 2n = 26 to 2n = 68. However, cytogenetic analyses have been conducted only to 1/3rd of the turtle species, often limited to conventional staining methods. In order to expand our knowledge of the karyotype evolution in turtles, we examined the topology of the (TTAGGG)_n_ telomeric repeats and the rDNA loci by fluorescence in situ hybridization (FISH) on the karyotypes of two emydids: the Sicilian pond turtle, *Emys trinacris,* and the yellow-bellied slider, *Trachemys scripta scripta* (family Emydidae). Furthermore, AT-rich and GC-rich chromosome regions were detected by DAPI and CMA_3_ stains, respectively. The cytogenetic analysis revealed that telomeric sequences are restricted to the terminal ends of all chromosomes and the rDNA loci are localized in one pair of microchromosomes in both species. The karyotype of the Sicilian endemic *E. trinacris* with diploid number 2n = 50, consisting of 13 pairs of macrochromosomes and 12 pairs of microchromosomes, is presented here for first time. Our comparative examination revealed similar cytogenetic features in *Emys trinacris* and the closely related *E. orbicularis*, as well as to other previously studied emydid species, demonstrating a low rate of karyotype evolution, as chromosomal rearrangements are rather infrequent in this group of turtles.

## 1. Introduction

The family Emydidae includes 53 species [1] of semi- or fully aquatic turtles, distributed mainly across North America and north of Mexico, except for few taxa present in Greater Antilles, Mexico, Central and South America (*Trachemys*) [2,3] and Europe (*Emys orbicularis* and *E. trinacris*) [4]. The turtles of the family Emydidae are divided in two subfamilies: Deirochelyinae and Emydinae [5]. The subfamily Deirochelyinae incorporates six genera and the majority of the emidid species, including one of the most invasive turtle species in the world, the red-eared slider *Trachemys scripta elegans.* The subfamily Emydinae includes only 11 species, including the turtles of the genus *Emys.* The polytypic European pond turtle *Emys orbicularis* (Linnaeus 1758), which is widely spread in Eurasia and the Maghreb, and the endemic Sicilian pond turtle, *Emys trinacris*, Fritz et al., 2005, are the only species belonging to the genus *Emys*, and the only Palearctic representatives of the predominantly Nearctic family Emydidae [6,7]. A third species has been proposed, namely *Emys blandingii* [1,8], but its taxonomic placement in the genus *Emys* or in the genus *Emydoidea* is still under debate [9,10]. *Emys orbicularis* and *Emys trinacris* are poorly distinguished according to traditional morphological or morphometric taxonomic characters and are often considered a “cryptic” species [11,12]. However, recent phylogenetic reconstructions based on genomic data revealed that these two emydid species are closely related, with *E. trinacris* being the sister to all the known mitochondrial lineages of *E. orbicularis* s.l. [11,13,14,15,16]. 

The cytogenetic analysis of emydid species is restricted mainly to Giemsa stained chromosomal preparations [17], with G-banding staining being applied in few cases [18,19,20,21,22,23,24]. Molecular cytogenetic methodologies were applied only recently to explore the karyotype evolution of emydid turtles, such as fluorescence in situ hybridization (FISH), with probes specific to the telomeric sequences (TTAGGG)_n_ [22,25,26,27] and the rDNA loci [20,27]. FISH is a technique that allows one to detect the presence and distribution of a sequence of interest directly on the chromosome metaphase of the studied species [28,29,30,31,32,33,34]. The comparative analysis of the in situ hybridization pattern, using a combination of probes specific for different genomic regions, can be informative for phylogenetic analysis [35,36]. The most common cytogenetic markers used for comparative FISH analysis are the telomeric motifs (TTAGGG)_n_, microsatellite markers and the rDNA loci [37,38,39,40,41,42,43,44]. The telomeric repeats can be located at terminal and interstitial regions of vertebrate chromosomes [45,46,47], can be lost or gained during the processes of karyotype evolution and can be potentially informative phylogenetic markers [39,40,46,47]. The 45S rDNA loci, comprising the 18S and 28S regions, usually form long tandem clusters in the chromosomes. The transcriptionally active 45S rDNA loci, often referred to as the nucleolus organizer regions (NORs), can be identified by silver staining (Ag-NOR), and both inactive and active rDNA loci can be detected accurately by fluorescence in situ hybridization (FISH) with specific probes [42]. 

In this study, we performed both conventional and molecular cytogenetic analysis to characterize the karyotype of the Sicilian endemic *Emys trinacris*, including karyotype reconstruction, DAPI, and CMA_3_ staining to detect AT-rich and GC-rich chromosome regions, and FISH with both probes for the (TTAGGG)_n_ telomeric repeats and for the rDNA loci. 

In addition, we decided to analyze the yellow-bellied slider *Trachemys scripta scripta* (Schoepff 1792). This is an often invasive species in Italy that poses as a direct competitor to the native turtles of the genus *Emys* [48]. We describe here for the first time the karyotype and the distribution of telomeric (TTAGGG)n repeats in this species.

For both species, we compared our results with the previously published data on *Emys orbicularis* [21], *Trachemys scripta elegans* (Wied 1838) and *Trachemys dorbigni* (Duméril and Bibron 1835) [19,20,25] in order to expand our knowledge on the karyotype evolution of emydid turtles.

## 2. Materials and Methods 

### 2.1. Studied Material

Peripheral blood was collected, in accordance with International and Institutional Ethical rules (Project ID: 2016-NAZ-0012, CUP: B72F16000130005), from the dorsal coccygeal vein with a heparinized sterile syringe, based on the protocol of Redrobe et al. [49] from two specimens of *E. trinacris* (ETR) and a single specimen of *T. s. scripta* (TSS) (Table 1).

### 2.2. Species Identification

*Emys trinacris* and *E. orbicularis* have similar external morphology and can be easily misidentified. In light of previous reports of introduced *Emys orbicularis* specimens in Sicily [15,50], we decided to verify the taxon identification of our specimens by sequencing and analyzing a fragment of the mitochondrial cytochrome b gene (for similar approach see Mazzoleni et al. [44,51]). Total DNA was isolated using the Real Genomics “Genomic DNA Extraction Kit” (RBC BioScience, New Taipei City 23145, Taiwan) following the manufacturer’s protocol. A fragment of the mitochondrial gene cytochrome b was amplified and sequenced following the protocol described by Marrone et al. [16]. 

*E. trinacris* and *E. orbicularis* cytochrome b (cytb) sequences from this study and other ones downloaded from GenBank were aligned and used as an input for Bayesian Inference phylogenetic reconstruction (BI) following the pipeline described by Belaiba et al. [52]. A cytb sequence of the emydid *Glyptemys muhlenbergi* was included in the analysis as an outgroup for rooting the tree.

### 2.3. Cytogenetic Examination

Chromosome suspensions were prepared from whole blood cell culture following the protocol of Dumas et al. [39] with some modifications. Briefly, up to 200 μL of whole blood were cultivated in 5 mL of RPMI medium (GIBCO, Thermo Fisher Scientific Waltham, MA USA) at 30 °C for 4–7 days. Three hours before harvesting, 40 μg of colchicine were added following a previous protocol [21]. 

The pattern of heterochromatin distribution was analyzed with CG-specific chromomycin A_3_ (CMA_3_) and 4′,6-diamidino-2-phenylindole (DAPI) sequential staining in ETR samples with the aim to detect, respectively, GC/AT rich regions as previously performed in *T. s. elegans* [20]. Amplification and hybridization of 45S rDNA probes labelled with biotin-dUTP were performed on ETR as reported by Mazzoleni and colleagues [41]. The distribution of the telomeric sequence (TTAGGG)_n_ was analyzed in ETR and TSS using in situ hybridization with a FITC-conjugated peptide nucleic acid (PNA) oligonucleotide probe (Panagene, Cambridge Research Biochemicals, Belasis Hall Technology Park Billingham, Cleveland TS23 4AZ UK). FISH experiments with telomeric probe were repeated twice, as post-hybridization washes were performed in high and low stringency conditions in order to accurately detect interstitial telomeric repeats (ITRs) following previous protocols [39,40,41]; in particular, we used 50% formamide and 2 x SSC at 37 °C for 20 min at low stringency, while we used 1 x PBS at 58 °C for 10 min at high stringency.

Images were captured using an Axio Zeiss microscope (equipped with a Zeiss digital camera). DAPI inverted banding and karyotype reconstruction were carried out for both ETR and TSS samples according to the protocols described by Dumas et al. [39]. Chromosome numbering for *E. orbicularis* followed Iannucci and colleagues [21]. The software Adobe Photoshop was used for figure preparation.

Moreover, in a wider perspective, we compared our data with those available for other emydid species, such as *T. s. elegans* and *Trachemys dorbigni* [20,25,26,53], in order to expand our knowledge of the karyotype evolution of emydid turtles.

## 3. Results and Discussion

**Figure 1:**
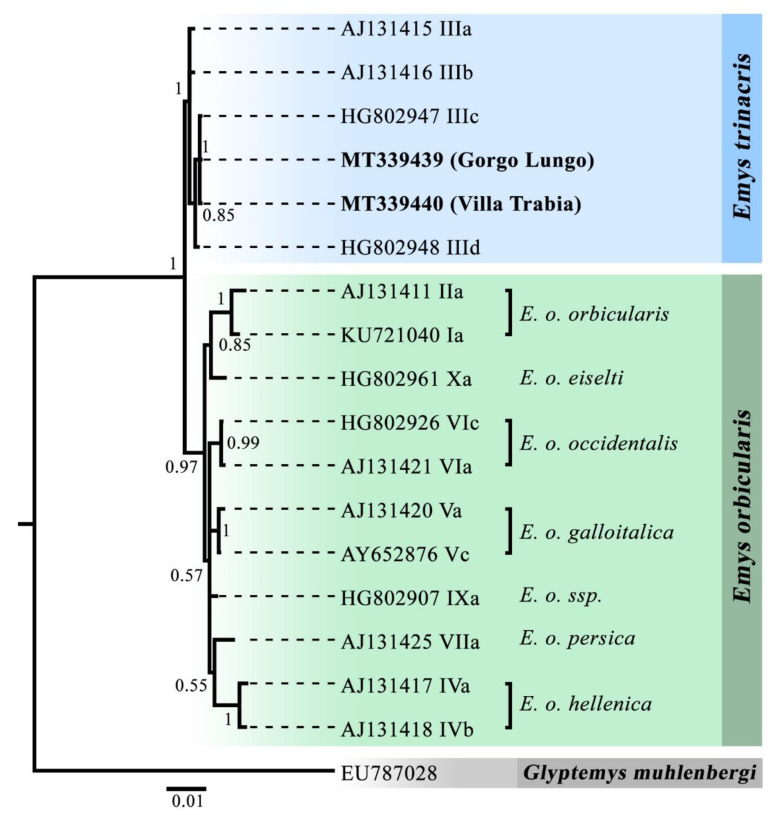
BI phylogenetic inference of *Emys orbicularis* and *E. trinacris* based on a 1012-bp long fragment of the mitochondrial gene cytb. For *E. orbicularis*, currently recognized subspecies are indicated. Numbers at nodes are Bayesian posterior probability values. GenBank accession numbers for previously published sequences are reported. Haplotype nomenclature follows Stuckas et al. [14]. The two novel *Emys trinacris* sequences are reported in bold.

Both our *E. trinacris* specimens share an identical cytb haplotype, which corresponds to the widespread Sicilian “lineage IIIc” according to the categorization of Vamberger et al. [15]. The BI phylogenetic reconstruction thus confirmed their identification as *E. trinacris* (Figure 1). Our sequences were deposit in GenBank under the accession numbers Gorgo Lungo: MT339439 and Villa Trabia: MT339440.

**Figure 2:**
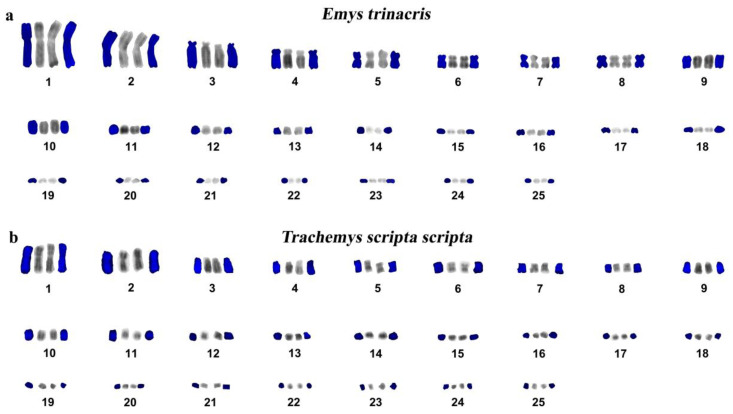
Reconstructed karyotypes of *E. trinacris* (**a**) and *T. s. scripta* (**b**); DAPI—blue and DAPI inverted chromosomes—grey for each pair of chromosomes homologues.

**Figure 3:**
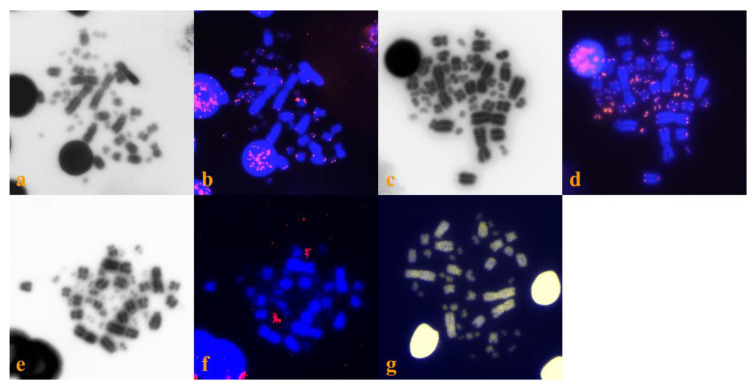
Topology of telomeric repeats in *Emys trinacris* (**a**,**b**) and in *T. scripta* (**c**,**d**). Topology of rDNA loci in *Emys trinacris* (**e**,**f**); DAPI-inverted metaphases permit a better visualization of chromosome morphology (**a**,**c**,**e**); hybridization signals of both telomeric (**b**,**d**) and rDNA (**f**) probes were pseudocolorized in red, while chromosomes were colored in DAPI blue. CMA_3_/DAPI staining overlapped in *Emys trinacris* (**g**).

Both *E. trinacris* specimens have the same karyotype with diploid chromosome number 2n = 50, consisting of 8 pairs of metacentric macrochromosomes, 5 pairs of acrocentric macrochromosomes and 12 pairs of acrocentric microchromosomes (Figure 2). The telomeric repeats were detected only at the terminal ends of all chromosomes (Figure 3b), while the rDNA loci were detected in a pair of microchromosomes (Figure 3f). CMA_3_ strongly stained regions rich in CG at centromeres, while DAPI did not stain (Figure 3g).

*T. s. scripta* specimen had a karyotype with 2n = 50 chromosomes consisting of 8 pairs of metacentric macrochromosomes, 5 pairs of acrocentric macrochromosomes and 12 pairs of acrocentric microchromosomes (Figure 2). Telomeric repeats were visible only at the terminal ends of all chromosomes (Figure 3d). 

Furthermore, we compared our results for *E. trinacris* and *T. s. scripta* with previously published cytogenetic data, specifically with *E. orbicularis*, *T. s. elegans* and *T. dorbigni* [20,21]. Both *E. trinacris* (this study) and *E. orbicularis* [21] share identical karyotypes, considering the chromosome morphology. In addition, identical patterns between the two species were also found for the distribution of the telomeric repeats, which are localized only at the terminal ends of chromosomes, and the distribution of the rDNA loci, which are localized in the first microchromosome pair of the complement ([21], this study). The comparison between the two species of the genus *Emys* shows that they have similar karyotypes, in agreement with previous features studied [11,12,13,16]. *T. s. scripta* showed a similar pattern with *E. trinacris* (this study) and *E. orbicularis* for all cytogenetic markers, suggesting once more the extreme karyotypic conservation of turtle families [22,54]; these data are in agreement with previous ones on repetitive sequence conservation, which are evidence of no occurrence of genome reorganization [39,46,47]. CMA_3_ staining in *Emys trinacris* (Figure 3g) showed GC content localized in the centromere and telomere in some chromosomes, in accordance with a previous report in *T. s. elegans* [53]. Additionally, it was previously shown that the chromosomes that carry the genes of the nucleolar organizing region (NOR) vary in the degree of heteromorphy and often correspond to the sex chromosomes in turtles [22,55]. Despite evidence from molecular phylogenetic studies that show differences between *E. trinacris* and *E. orbicularis* [13,56], our cytogenetic comparative analysis revealed similarity between the two species.

We also compared the karyotypes of *T. s. scripta*, *T. s. elegans* and *Trachemys dorbigni*. The analysis showed that these taxa have identical diploid numbers with 2n = 50 chromosomes, and karyotypes with 8 pairs of meta/submetacentrics, 5 pairs of acrocentrics and 12 pairs of microchromosomes (Figure 2b). In the same context, DAPI-inverted karyotype of *T. s. scripta* (Figure 2b) showed a similar banding pattern with the previously published G/DAPI stained karyotype of *T. s. elegans* and *Trachemys dorbigni* [20,26]. Furthermore, *T. s. scripta*, *T. s. elegans* and the closely related *T. dorbigni* share identical pattern for the topology of the telomeric repeats, restricted to terminal topology of all chromosomes [20,22,25]. This is in good accordance with the low level of molecular differentiation recently revealed for the subspecies of *Trachemys scripta* [56].

From a broader perspective, despite the fact that turtles in general have an extensive variability in chromosome numbers across species, ranging from 2n = 26 to 2n = 68 [22], emydids are rather conserved in the repetitive sequence distribution. Emydids seem to have similar karyotypes based on chromosome morphology, and their diploid chromosome numbers vary from 2n = 48 to 2n = 52, with 2n = 50 chromosomes (26 macro- and 24 microchromosomes) being the most common [17]. Across five studied emydid species, the expected terminal topology of telomeric repeats was reported in *Chrysemys picta* [27], *E. orbicularis* [21], *T. s. elegans* [25] and *T. dorbigni* [25], while interstitial telomeric repeats were detected only in the centromeric region of chromosome 9 of *Glyptemys insculpta* [22]. 

## 4. Conclusions

Despite evidence from molecular phylogenetic studies that show differences between *E. trinacris* and *E. orbicularis*, our cytogenetic comparative analysis reveals striking similarity of the karyotypes between the two species. The conserved diploid chromosomal number, the similarities in chromosome morphology and the lack of interstitial telomeric repeats indicate that chromosomal rearrangements are rather infrequent, supporting the view of a conservative genome organization and an extremely low rate of karyotype evolution in emydid turtles.

## Figures and Tables

**Table 1:** List of the samples analyzed in the frame of this study.

Latin Name	Code	Samples	Specimens
*Emys trinacris*	ETR	blood	male collected in a natural pond (Gorgo Lungo, WGS84 geographical coordinates: 37.901131 N, 13.408438 E; altitude: 890 m a.s.l.)
		blood	male collected in an ornamental basin of a public garden within the town of Palermo (Villa Trabia, WGS84 geographical coordinates: 38.129757 N, 13.347749 E; altitude: 20 m a.s.l.).
*Trachemys scripta scripta*	TSS	blood	female collected at the Botanical Garden of the University of Palermo (Italy)
